# Depression and COVID-19 patients with chronic pain: practical considerations

**DOI:** 10.1186/s41983-021-00286-4

**Published:** 2021-02-24

**Authors:** A. Alcántara Montero, S. R. Pacheco de Vasconcelos, F. Peñato Tarifa

**Affiliations:** 1 Manuel Encinas Health Center, Doctor Rodríguez de Ledesma Avenue, 8, 10002 Cáceres, Spain; 2 Complejo Hospitalario Universitario de Cáceres, Hospital Universitario, Cáceres, Spain

**Keywords:** Coronavirus, SARS-CoV-2, COVID-19, Chronic pain, Antidepressants

## To the Editor,

### Introduction

We read with interest the literature review by Widyadharma and colleagues on pain as clinical manifestations of coronavirus disease 2019 (COVID-19) and its management in the pandemic era. The authors highlight the association of psychiatric disorders such as depression to pain in these patients, which leads to a significant decrease in the quality of life [1].

### Main text

The pandemic brought about by COVID-19 has resulted in the need for the public to isolate or “maintain social distance” to prevent the spreading of the disease. A survey carried out by the Spanish Pain Society on 340 participants shows that 91.4% of people with chronic pain believe that the lockdown has affected their emotional state, and 63% say that their quality of sleep is worse than before the pandemic [2].

Pain as a cause of stress and its sensory and emotional dimensions are widely connected and there are several factors, whether psychological, social, or neurobiological, which determine this circle, which is often so difficult to break unless a biopsychosocial therapeutic approach is used [3, 4]. We also reflect on whether the stress caused by this situation, the anxiety, and even the resulting depression negatively contribute by closing this vicious circle, which undoubtedly is the case [5]. In fact, patients with chronic pain who must, just like any other citizen, be in isolation or observe social distancing due to the COVID-19 pandemic may experience a decline in their physical and emotional well-being [6].

In their review, the authors also make reference to the use of opioids, non-steroidal anti-inflammatory drugs, and corticosteroids in patients with COVID-19 [1]. Taking into account the assumptions made, are any special recommendations necessary regarding the use of antidepressants in these patients? We are aware that most clinical practice guidelines concur that tricyclic antidepressants (amitriptyline) and serotonin and noradrenaline reuptake inhibitors venlafaxine and duloxetine are first-line drugs for the treatment of peripheral neuropathic pain [7], which may also be present in patients with COVID-19 [1]. In the event that the patient is undergoing treatment with drugs for COVID-19 which prolong the QTc interval, normally under clinical trial, we must check that they do not interact with the antidepressants. For example, amitriptyline and venlafaxine may cause QTc interval prolongation, but currently, evidence is lacking on any risk of Torsades de Pointes when they are administered at the recommended dose. For this reason, we recommend monitoring using an electrocardiogram in the event of a combination or considering an alternative such as duloxetine [6]. It is also necessary to be aware of the possible interactions of these analgesic and adjuvant drugs with the pharmacological treatment applied to patients who are COVID-19 positive and affected by the illness. With regard to the latter, while these clinical and therapeutic dilemmas are resolved, specific information can be found on these medical interactions at trustworthy sites, such as https://www.hep-druginteractions.org/checker [6].

### Conclusions

It unfortunately seems quite likely that COVID-19 is here to stay, so we must ensure that all necessary means are set in motion so that the care of chronic pain sufferers is not undermined.

## Data Availability

Data is available whenever requested.

## Abbreviation

COVID-19: Coronavirus disease 2019

## References

[1] Widyadharma IPE, Sari NNSP, Pradnyaswari KE, Yuwana KT, Adikarya IPGD, Tertia C (2020). Pain as clinical manifestations of COVID-19 infection and its management in the pandemic era: a literature review. Egypt J Neurol Psychiatr Neurosurg.

[2] [Covid-19 chronic pain patients survey results] [Internet] [Accessed 5 Jan 2021]. Available from: https://www.sedolor.es/download/resultado-encuesta-pacientes-dolor-cronico-covid-19/

[3] Dueñas M, Ojeda B, Salazar A, Mico JA, Failde I (2016). A review of chronic pain impact on patients, their social environment and the health care system. J Pain Res.

[4] Solé E, Racine M, Tomé-Pires C, Galán S, Jensen MP, Miró J (2020). Social factors, disability and depressive symptoms in adults with chronic pain. Clin J Pain.

[5] Blackburn-Munro G, Blackburn-Munro RE (2001). Chronic pain, chronic stress and depression: coincidence or consequence?. J Neuroendocrinol.

[6] Alcántara Montero A (2020). Pacheco de Vasconcelos SR. [COVID-19 and chronic pain: many questions and few certainties]. Semergen..

[7] Alcántara Montero A, Ibor Vidal PJ, Alonso Verdugo A, Trillo CE (2019). Update in the pharmacological treatment of neuropathic pain. Semergen..

